# Differential transmissibility to *Anopheles arabiensis* of *Plasmodium vivax* gametocytes in patients with diverse Duffy blood group genotypes

**DOI:** 10.1186/s12936-023-04570-x

**Published:** 2023-04-25

**Authors:** Andargie Abate, Jifar Hassen, Laurent Dembele, Didier Menard, Lemu Golassa

**Affiliations:** 1 Aklilu Lemma Institute of Pathobiology, Addis Ababa University, Addis Ababa, Ethiopia; 2 College of Medicine and Health Sciences, Bahir Dar University, Bahir Dar, Ethiopia; 3 School of Applied Natural Science, Adama Science and Technology University, Adama, Ethiopia; 4 Malaria Research and Training Centre (MRTC), Université des Sciences, des Techniques et des Technologies de Bamako (USTTB), Bamako, Mali; 5 Institut Pasteur, Malaria Genetics and Resistance Unit, Université Paris Cité, INSERM U1201, 75015 Paris, France; 6 Federation of Translational Medicine, Institute of Parasitology and Tropical Diseases, University of Strasbourg, UR7292 Dynamics of Host-Pathogen Interactions, 67000 Strasbourg, France; 7 Laboratory of Parasitology and Medical Mycology, Strasbourg University Hospital, Strasbourg, France

**Keywords:** Duffy blood group, *Anopheles* mosquitoes, *Plasmodium vivax*, Membrane feeding assay, Ethiopia

## Abstract

**Background:**

Measuring risk of malaria transmission is complex, especially in case of *Plasmodium vivax*. This may be overcome using membrane feeding assays in the field where *P. vivax* is endemic. However, mosquito-feeding assays are affected by a number of human, parasite and mosquito factors. Here, this study identified the contributions of Duffy blood group status of *P. vivax*-infected patients as a risk of parasite transmission to mosquitoes.

**Methods:**

A membrane feeding assay was conducted on a total of 44 conveniently recruited *P. vivax* infected patients in Adama city and its surroundings in East Shewa Zone, Oromia region, Ethiopia from October, 2019 to January, 2021. The assay was performed in Adama City administration. Mosquito infection rates were determined by midgut dissections at seven to 8 days post-infection. Duffy genotyping was defined for each of the 44 *P. vivax* infected patients.

**Results:**

The infection rate of *Anopheles* mosquitoes was 32.6% (296/907) with 77.3% proportion of infectious participants (34/44). Infectiousness of participants to *Anopheles* mosquitoes appeared to be higher among individuals with homozygous Duffy positive blood group (TCT/TCT) than heterozygous (TCT/CCT), but the difference was not statistically significant. The mean oocyst density was significantly higher among mosquitoes fed on blood of participants with FY*B/FY*B^ES^ than other genotypes (P = 0.001).

**Conclusion:**

Duffy antigen polymorphisms appears to contribute to transmissibility difference of *P. vivax* gametocytes to *Anopheles* mosquitoes, but further studies are required.

## Background

Malaria continues to be one of the most devastating parasitic diseases that affect humans, having a significant impact on global public health and the economy. According to World Health Organization (WHO) estimates, there were 247 million cases and 619,000 fatalities in 2021 [1]. The disease is caused by four different human malaria causing species (*Plasmodium falciparum, Plasmodium vivax, Plasmodium malariae* and *Plasmodium ovale*), with *P. vivax* being the most widespread extending beyond the limits of *P. falciparum*. In endemic regions where *P. vivax* and *P. falciparum* co-exist, *P. vivax* continues to be the main cause of malaria because its incidence declines more slowly than that of *P. falciparum*. *Plasmodium vivax* causes severe and fatal outcomes which has reversed the historic notion of benign of *P. vivax* infections [2]. However, despite this burden, *P*. *vivax* does not attract as much attention as *P. falciparum,* particularly in sub-Saharan Africa (SSA) [3].

In recent years, there has been an interest in blocking the transmission of malaria parasites from humans to mosquitoes in order to control, and finally, eliminate the disease [4]. However, measuring risk of infection, and evaluating the efficiency of transmission-blocking activities are complex [5], especially in case of *P.vivax* due to inherent biological hurdles of in vitro culture system [3, 6]. These make the mechanisms involved in the transmission of the parasite from human to mosquito to be poorly understood, but this uncertainty may be overcome using mosquito feeding assays in the field where *P. vivax* is endemic [7].

Indeed, infectiousness of gametocytes to mosquitoes are mediated by a number of human, parasite and mosquito factors including density, maturity and sex ratio of gametocytes [7–9], mosquito immunity [10, 11] and parasite interactions with microbiota in the midgut [11, 12]. The other factor that may hinder gametocyte infectiousness is naturally acquired human immunity affecting density and development of both asexual and sexual parasites [8]. Duffy antigen blood group system is one natural resistant mechanism in which Duffy-negative people were considered naturally resistant to *P. vivax* infection [13], although the dogma has been challenged in recent studies [14–17].

Duffy antigen blood group was poorly studied in countries where the disease is endemic despite its crucial role, but with a recent interest in malaria elimination, the number of studies of the Duffy antigen has increased. The scientific paradigm is that Duffy antigen also called Duffy antigen receptor for chemokines (DARC) or the FY gene is required as a receptor for *P. vivax* merozoites to invade erythrocytes, however it is in question in Africans [14, 18, 19] suggesting that the parasite may utilize alternative receptors.

The Duffy antigen is characterized by three main alleles: FYA, FYB and FYO. Genetic polymorphisms were identified in humans influencing the expression of the Duffy antigen [20], and thereby modulating the susceptibility and the naturally-acquired immune response to vivax malaria. The susceptibility varied based on the associated Dufy blood group antigens (Fy^a^ and Fy^b^) [21]. This has explained that malaria parasite transmission has been associated to gene selective pressure in the human genome [22] affecting the human susceptibility to mosquitoes.

Therefore, the utilization and evaluation of transmission-reducing activities require a better understanding of the human reservoir of infection. This is because understanding the human genetic factors explaining why some people are more infectious than others may provide clues for the development and evaluation of new strategies to control and eliminate malaria. Thus, this study was aimed to determine whether Duffy genotypes impact *P. vivax* malaria transmission in East Shewa Zone, Oromia region, Ethiopia.

## Methods

### Study area and period

The study was conducted in Adama city administration, and its surroundings, East Shewa Zone, Oromia region, Ethiopia from October 2019 to January 2021 as described previously [23]. Adama which is located 100 kms far from Addis Ababa, the capital city of Ethiopia, is 1623 m above sea level, with an annual temperature of 20.5 °C and an annual precipitation of 808 mm. The surrounding health centres were located within a 25 km radius of Adama city. Adama City and its surrounding areas were located in the Great Rift-Valley and characterized by seasonal malaria where both *P. falciparum* and *P. vivax* co-exist. *Plasmodium vivax* malaria is the dominant one in the area [24]. Thus, the current study was carried on febrile patients recruited from public health institutions (Adama malaria diagnostic centre, 7 health centres in the Adama City Administration and 5 health centres from surrounding areas). *Plasmodium vivax* positive patients were transported to the membrane feeding assay centre. The membrane feeding assay was performed at Adama public health research and referral laboratory insectary centre, Adama City administration.

### Study population

The study populations were patients who had self-reported febrile illness, visited selected health institutions in Adama City Administration and its surroundings, as well as microscopically confirmed *P. vivax* positive patients during the study period.

### Inclusion criteria

Those febrile patients with uncomplicated vivax malaria aged 5 years and older were included in the study irrespective of gametocyte screening to include all potentially infectious individual because of limitation of microscopy to detect gametocytes.

### Exclusion criteria

Patients with age < 5 years old, *P. falciparum* mono-infection, severe *P. vivax* malaria, pregnant women, and had history of anti-malarial drugs within one month were excluded from this study.

### Sample size determination and sampling procedure

The membrane feeding assay is logistically complicated and expensive, and as such many factors were considered to be possible sources of variability in the assay. As a result, the sample size was determined based on the number of patients conveniently visited the selected health institutions and microscopically confirmed *P. vivax* infection, and availability of mosquitoes within the study period. A total of 44 *P. vivax* infected patients were recruited to the study in order to determine the transmission variation across Duffy antigen difference.The health facilities were purposively selected because of their proximity to Adama insectary centre where the membrane feeding assay was conducted. And then, all microscopically confirmed *P. vivax* positive patients during the study period were recruited to study conveniently.

### Detection of *Plasmodium* infection

Patients who visited the selected health centres for malaria diagnosis and treatment were asked to provide finger prick blood samples. After cleaning their finger surfaces with alcohol-soaked sterile cotton, blood samples were collected from their finger pricks, and both thin and thick blood films were prepared in a single slide labeled with the patients' identification number and date of collection. According to the protocol, the thin smear was fixed with methanol, and the thick and thin blood smears were stained with 2% Giemsa solution [25]. Two skilled microscope experts blinded to each other's results independently examined each dried blood film under a 100 × oil immersion microscope. Third reader was used to read slides with conflicting results blindly, and the majority of their readings were considered the final result. The formula (Number of parasites counted X8000)/(Number of counted WBCs) was used to calculate the parasite density by considering 8000 WBCs/μL. The parasitaemia was then determined by averaging the readings from the two independent readers.

### Blood samples collection and processing

Patients were recruited and transported to mosquito infection laboratory at Adama public health research and referral laboratory insectary centre once *P. vivax* infection was confirmed by microscopy at each health centre. Following their arrival, each study participant had provided approximately 5 ml of venous blood. The collected blood samples were divided for the mosquito membrane feeding assay and molecular analysis. For membrane feeding assay, 3 ml blood sample was collected in heparin-containing tube, while the remaining 2 ml blood sample was collected in EDTA tube. Immediately after collection, EDTA blood samples were spotted onto Whatman 3 MM filter paper as previously described [26, 27] with some modifications. For each participant, two to four blood spots, near to ~ 60 μl each spot, were collected for dried blood spots (DBS) which were then dried and stored at room temperature until use.

### *Plasmodium* species identification by quantitative real-time PCR (qPCR)

The genomic DNA was extracted using the QIAamp DNA Mini blood kit based on the manufacturer’s protocol, with some modifications. In each tube, 1 mL of PBS (1X)/saponin (0.5%), 180 µL of buffer ATL, 20 µL proteinase K, 200 µL of Buffer AL, 200 µL of ethanol (96–100%), 500 µL of Buffer AW1, 500 µL of Buffer AW2, and 200 µL of elution buffer (AE buffer) were added. The manufacturer-recommended incubation temperature, time, and centrifugation rate were used to carry out the extraction. The extracted DNA was then placed in 1.5 mL Eppendorf tubes, which were then stored in a freezer at − 20 °C until use.

Real-time PCR amplification of species-specific segments of the *cytochrome b* described previously [28] was performed to confirm pure and mixed *P. vivax* infection with *P. falciparum*. The forward and reverse nucleotide primers for *P. vivax* were 5′-TGCTACAGGTGCATCTCTTGTATTC-3′, and reverse-5′-ATTTGTCCCCAAGGTAAAACG-3′, respectively, and 5′-ATGGATATCTGGATTGATTTTATTTATGA-3′, reverse-5′-TCCTCCACATATCCAAATTACTGC-3′ for *P. falciparum*, respectively. The PCR amplifications were carried out with a final volume of 20 μL, 4 μL of Evagreen HRM Mix (5x), 0.5 μL of each primer, and 5 μL of DNA template using the CFX96TM Real-Time PCR detection system (Bio-Rad Laboratories Ltd). The thermal condition was 94 °C for 15 min, 20 cycles of 30 s at 94 °C, 1 min at 58 °C for, 72 °C for one and a half min and 10 min at 72 °C followed by a 20 °C cooling step. Each PCR run using water as negative, and *P. vivax* DNA from blood sample with parasite density estimated ~ 8000 parasites/µL and *P. falciparum* DNA at 5 ng/µL as positive control. The qPCR Ct value was calculated based on a threshold set at 200 for each sample tested positive for *P. vivax*.

### Duffy genotyping

Duffy genotyping was conducted by PCR amplification from *P. vivax* human isolates as described before [23, 29]. Each PCR contained 33 μL molecular water, 1 μL of each primer, 10 μL of 5X HOT blend Taq polymerase (Sotis biodyne), and 5 μL of DNA template for a total of 50 μL volume. The mixture was denatured for 15 min at 94 °C followed by 40 cycles each at 94 °C for 20 s, 60 °C for 20 s, 72 °C for 1 min, and final elongation for 10 min at 72 °C. The primers for GATA-1 transcription factor binding site of the FY gene and exon 2 of the DARC gene were described previously [23]. The PCR products were purified by Agencourt AMPure XP bead-based purification, and then the products were sent to Biofidal (France) for sequencing using Sanger sequencing. The CLC Genomics Workbench 22.0 (Qiagen, Germany) software was used to analyze the sequenced nucleotides on both strands. The sequences generated in this study were aligned with their respective reference sequences.

### Mosquito membrane feeding assay

Mosquito membrane feeding assay was conducted using colonies of *Anopheles arabiensis* that were reared in Adama public health research and referral laboratory insectary. The insectary is located at malaria diagnostic centre in Adama city administration, East Shewa zone, Oromia region, Ethiopia. *Anopheles arabiensis* mosquitoes were reared at 24–27 °C temperature and relative humidity of 70–90% maintained conditions.

Starved female *Anopheles* mosquitoes were selected by mouth aspirator, and distributed into paper cups (40 mosquitoes per cup) covered with a net. The glass membrane feeder with Parafilm was attached with thermo-regulating water-bath machine maintaining water at 37 °C. The venous blood collected in heparinized tube was immediately filled to glass feeder, and mosquitoes were allowed to feed through a Parafilm for 30 min to 1 h in a dark place. A constant 37 °C circulating water system was maintained to prevent exflagellation of the microgametocytes. 1 h post feeding, non-blood fed mosquitoes were removed with an aspirator and placed into another cup to kill them, while the blood fed mosquitoes were transferred into a big mosquito rearing cages and maintained under optimum biosafety standards until dissection. Sucrose solution (10%) impregnated cotton wool was put on each cage and replaced daily until dissection period as described earlier [30].

### Determination of mosquito’s infection status

After seven to 8 days post blood feeding, the mosquitoes were placed in a − 20 °C freezer for 10 min to immobilize them. Using 1% drop of mercurochrome solution on the microscope slide, midgut dissection was performed under a stereo-microscope, and then the dissected mid-gut with mercurochrome was covered with coverslip. Mosquito midgut infection was examined microscopically at 20 ×magnification by detecting oocysts. The number of oocysts was counted and recorded for each individual mosquito following standard protocol [30]. The presence and loads of oocysts were determined using microscopy with camera by projecting, and capturing the image into desktop (computer) manually.

### Statistical analysis

The data were entered into Epi-Data version 3.1 and exported to Statistical Package for Social Sciences (SPSS) version 25 for analysis. Descriptive statistics were used to describe the frequencies of mosquito infection, Duffy genotypes, phenotypes, and other related variables using figures and tables. All the graphs were drawn using GraphPad Prism software version 8.0. The differences in oocyst level related to Duffy antigen polymorphism were determined. P ≤ 0.05 was considered as statistically significant in this study.

### Ethical clearance

Ethical approval was obtained from Institutional Review Board (IRB) of Addis Ababa University (AAU), Aklilu Lemma Institute of Pathobiology (ALIPB), and National Research Ethics Review Committee. Study participants, their parents, or guardians received thorough explanations of their rights, purpose and procedures of the study. The participants were informed that the procedure might cause minor pain at the site of the blood drawing. The participants in the study received treatment in accordance to national treatment guidelines. The participants and/or their guardians were given the right to refuse or withdraw from the study at any time during the study. All participants were assured of confidentiality throughout the study.

## Results

### Description of the study participants

In total, the blood samples collected from conveniently enrolled 44 symptomatic *P. vivax* infected patients were fed to the laboratory-reared *Anopheles* mosquitoes via artificial membrane feeding from October 2019 to January 2021. Majority (75%) of study participants were males, and recruited from urban (Adama city administration) in residence (68.2%). Near to forty three percent of the study participants were in the age group of 25–44 years old. More than half (52.3%) of the participants were ever married (married, separated and divorced) (Table 1).

**Table 1:** Demographic characteristics of study participants in East Shewa Zone, Oromia region, Ethiopia

Variables	Category	Number (%)
Sex	Male	33 (75.0)
	Female	11 (25.0)
Age	< 24	16 (36.4)
	25–44	19 (43.2)
	> 45	9 (20.4)
Marital status	Never married	21 (47.7)
	Ever married	23 (52.3)
Residence	Urban (Adama City)	30 (68.2)
	Rural (Out of Adama)	14 (31.8)

### Frequency of mosquito infection by *Plasmodium vivax*

Amongst the 44 feeding assays carried out, the overall engorged feeding percentage of the laboratory-reared *Anopheles* mosquitoes was 63.3% (5257/8300 exposed mosquitoes). On average, 21 mosquitoes (range: 5–60; IQR: 10–28) were dissected per assay with a total of 907 mid-gut dissected mosquitoes. Near to thirty three percent (32.8%) of mid-gut dissected mosquitoes were infected with oocysts. Detailed information on the number of dissected and infected mosquitoes is given in Table 2. As determined by microscopically detected oocysts, the proportion of infectiousness of human to mosquitoes was found to be 77.3% (34/44 feeding assays) (Table 2).

**Table 2:** Characteristics details of mosquitoes exposed for membrane feeding assay in East Shewa Zone, Oromia region, Ethiopia

Characteristics	Frequency
Age (days) range of exposed mosquitoes (Mean ± SD)	2–8 (4.45 ± 1.27)
Average starvation period(h) ± SD (range)	12 ± 3.63 (4–20)
Total number of exposed mosquitoes	8300
Average number of exposed mosquitoes	160 (ranged 40 to 550) per experiment
% (no. with characteristic/no. tested) of: mosquito blood feeding rates	63.3% (5257/8300)
Infectious individuals	77.3% (34/44)
Infected mosquitoes	32.6% (296/907)

### Duffy blood group genotyping, parasitaemia, gametocytaemia and mosquito infection

#### Duffy blood group genotyping

Duffy genotyping was successfully performed on 44 blood samples collected from *P. vivax* infected patients. The majority (68.2%) of the study participants had heterozygous Duffy blood group (TCT/CCT). Duffy genotype FY*B/FY*B^ES^ (36.4%) was the most common followed by FY*A/FY*B^ES^ genotypes (29.5%). The most common phenotypes detected in the study were Fy (a − b +) and Fy (a + b − ) observed from 45.5% and 38.6% of participants, respectively (Table 3).

**Table 3:** Genotypes and phenotypes frequencies of Duffy blood group among *P. vivax* infected patients in East Shewa Zone, Oromia region, Ethiopia

Variables	Category	Frequency (%)
GATA Box polymorphism	TCT/CCT	30 (68.2)
	TCT/TCT	14 (31.8)
Genotype	FY*B/FY*B^ES^ FY*A/FY*B^ES^ FY*A/FY*B FY*B/FY*B	16 (36.4) 13 (29.5) 6 (13.6) 4 (9.1)
	FY*A/FY*A	4 (9.1)
	FY*B^ES^/FY*X	1 (2.3)
Phenotype	Fy(a − b +)	20 (45.5)
	Fy(a + b − )	17 (38.6)
	Fy(a + b +)	6 (13.6)
	Fy(a − b + ^weak^)	1 (2.3)

#### Parasitaemia and gametocytaemia

The median (range) of *P. vivax* parasite density was 6188(419–42,833) parasites/µL, while the median (range) gametocytaemia was 80 (0–2785) gametocytes/µL (Data not shown). *Plasmodium vivax* parasitaemia and gametocytaemia were not significantly different between Duffy blood groups although heterozygous Duffy blood group patients had a reduced mean *P. vivax* parasitaemia and an increased mean *P. vivax* gametocytaemia compared to homozygous Duffy blood group patients (Table 4).

**Table 4:** Parasitaemia and gametocytaemia level according to Duffy blood group among *P. vivax* infected patients in East Shewa Zone, Oromia region, Ethiopia

Variables	Category	Sample size	Mean parasites/µL	P value	Mean gametocytes/µL	P value
GATA Box polymorphism	TCT/CCT	30	6540	0.27	297	0.31
	TCT/TCT	14	9232		121	
Genotype	FY*B/FY*B^ES^	16	6672	0.30	294	
	FY*A/FY*B^ES^	13	6758		324	
	FY*A/FY*B	6	6487		104	0.93
	FY*B/FY*B	4	6572		136	
	FY*A/FY*A	4	16,009		133	
	FY*B^ES^/FY*X	1	1618		0	
Phenotype	Fy(a − b +)	20	6651		262	
	Fy(a + b − )	17	8935	0.68	279	0.88
	Fy(a + b +)	6	6487		104	
	Fy(a − b + ^weak^)	1	1618		0	

#### Mosquito infection

Mosquito infectivity was estimated qualitatively (presence of at least one mosquito positive with oocyst) and quantitatively (average number of oocysts per dissected mosquito).

#### Qualitative assessment

No significant difference was detected between the blood of patients, that led to the presence of at least one mosquito positive with oocyst, including the Duffy blood group, as shown in Table 5. The proportion of mosquito batches containing at least one mosquito positive with oocyst was higher in homozygous Duffy positive individuals (TCT/TCT, N = 30) than heterozygous (TCT/CCT, N = 14) although the difference was not statistically significant. Study participants with FY*B/FY*B (N = 4) and FY*A/FY*B (N = 6) genotypes were more infectious to *Anopheles* mosquitoes (100% in both groups). The proportion of mosquito batches containing at least one mosquito positive with oocyst was relatively higher in participants aged 25–44 years, ever married and rural residents (Table 5).

**Table 5:** Presence of at least one mosquito positive with oocyst by characters of participants in East Shewa Zone, Oromia region, Ethiopia

Variables	Category	Having at least one mosquito with Oocyst (s)		Fisher’s Exact
		No (%)	Yes (%)	
Sex	Male	8 (24.2)	25 (75.8)	0.68
	Female	2 (18.2)	9 (81.8)	
Age	= < 24	5 (31.3)	11 (68.8)	0.26
	25–44	2 (10.5)	17 (89.5)	
	= > 45	3 (33.3)	6 (66.7)	
Marital status	Never married	6 (28.6)	15 (71.4)	0.48
	Ever married	4 (17.4)	19 (82.6)	
Residence	Urban	10 (33.3)	20 (66.7)	**0.02**
	Rural	0	14 (100)	
GATA Box	TCT/TCT	2 (14.3)	12 (85.7)	0.46
	TCT/CCT	8 (26.7)	22 (73.3)	
Duffy genotype	FY*B/FY*B	0	4 (100)	0.07
	FY*A/FY*A	2 (50.0)	2 (50.0)	
	FY*A/FY*B	0	6 (100)	
	FY*B/FY*B^ES^	3 (18.8)	13 (81.3)	
	FY*B^ES^/FY*X	1 (100)	0	
	FY*A/FY*B^ES^	4 (30.8)	9 (69.2)	

Bold indicates statistically significant

#### Quantitative assessment

**Fig. 1:**
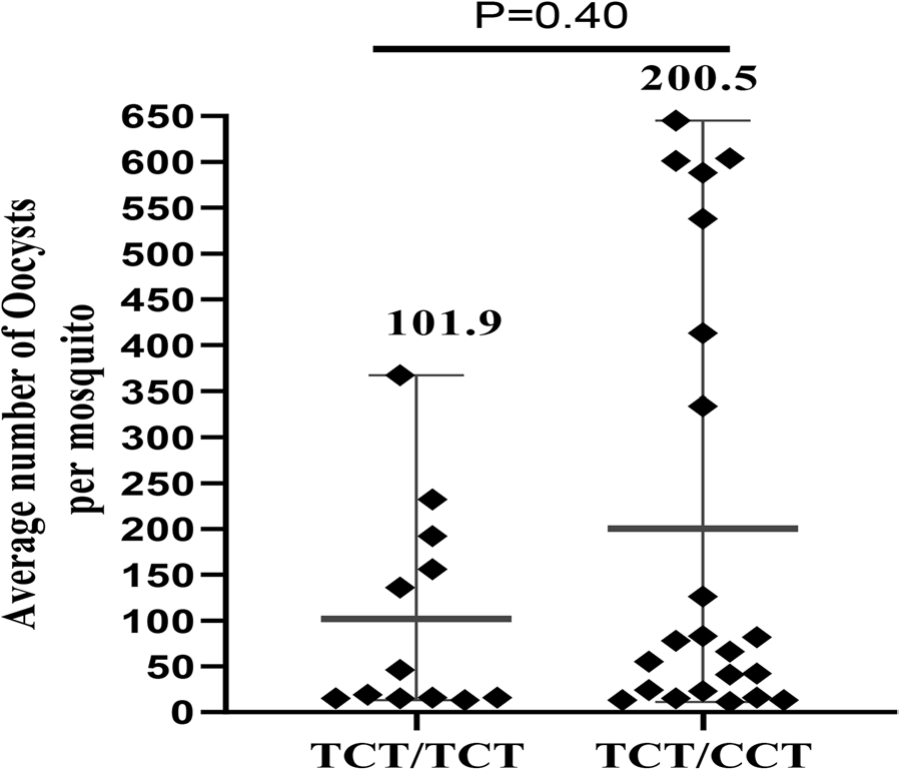
Comparison of oocyst levels between Duffy-negative and Duffy-positive symptomatic patients in East Shewa Zone, Oromia region, Ethiopia

**Fig. 2:**
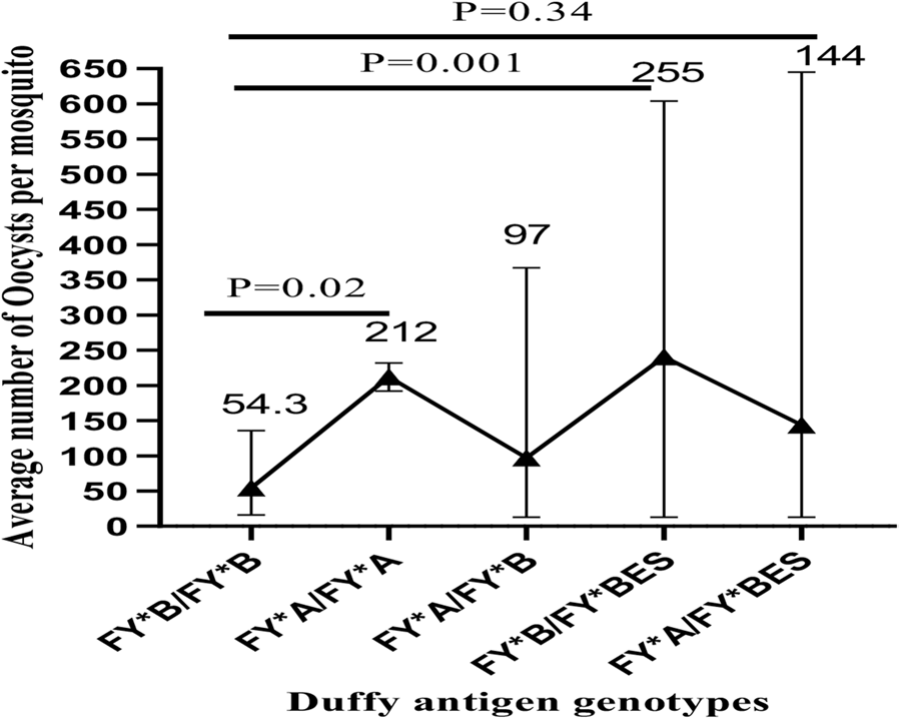
Relationship between Duffy blood group genotypes and oocyst densities per mid-gut dissected mosquitoes in East Shewa Zone, Oromia region, Ethiopia

The oocyst density in heterozygous Duffy positive individuals (TCT/CCT) was found to be relatively higher than homozygous Duffy-positive individuals (TCT/TCT) (Mean = 200.5 and 101.9, respectively) (Fig. 1) although the difference was not statistically significant (*P* = 0.34). However, the mean oocyst load of mid-gut dissected mosquitoes fed blood samples with FY*B/FY*B^ES^ genotype was significantly higher than those with FY*B/FY*B genotype (P = 001). The FY*B/FY*B^ES^ and FY*A/FY*B^ES^ showed a greater range of oocyst loads than other genotypes, in general (Fig. 2).

## Discussion

The proportion of infective mosquitoes is the most important outcome measure to determine human-mosquito infectiousness and for evaluating the impact of TBIs on human infectivity. Thus, the present study showed that more than half (77.3%) of *P. vivax* infected patients were infectious to mosquitoes, which is in line with findings in Colombia (87.4%) [31]. Given the difficulty and expense of the membrane feeding assay, a thorough understanding of the factors affecting mosquito infection would enable determination of the relative contributions of various groups to transmission.

This is the first study that gave an insight on the contribution of Duffy blood group system on the transmission of *P. vivax* to *Anopheles* mosquitoes. The current study revealed that those study participants with homozygous Duffy positive blood groups (TCT/TCT) were more infectious to mosquitoes than those with heterozygous Duffy positives (TCT/CCT) even if the difference was not statistically significant. This could be because Duffy positive individuals had higher *P. vivax* parasitaemia than heterozygous Duffy positives in the current study, which is consistent with other studies [23, 32]. This link could be due to the mosquito infections being directly related to parasite density as previously described [33–35]. However, the present study found that while gametocytaemia levels were higher in heterozygous Duffy positives, mosquitoes infection rates were lower.

The aforementioned explanations took into account a number of factors besides parasite densities that affect the frequency of mosquito infections, including sex ratio and gametocyte maturation of the parasite, blood volume, mosquito age, the interval between blood collection and mosquito feeding, and the degree of host immunity to the parasite [7] even though this study did not specifically address these factors. Furthermore, since previous research demonstrated that these human factors affected mosquito capacity and, consequently, oocyst survival, human host factors like diet and nutritional status could affect *Plasmodium* transmission to mosquitoes [36] which needs further investigation.

In this study, individuals with genotypes FY*B/FY*B and FY*A/FY*B were more infectious to *Anopheles* mosquitoes than individuals with other genotypes. It might be related with the fact that homozygous Duffy antigen positives were less likely to develop immune response against *P. vivax* blood-stage antigens, which could be due to the increased parasite burden and parasite-mediated immune-suppression as described previously. Moreover, people with high DARC expression are less likely to have antibodies against DBP. For instance, anti-MSP1 and anti-DBP antibodies were more likely developed in people with low DARC expression [37]. This is strongly supported by the observation that the human host’s naturally developed immunity influences the production of gametocytes from their asexual precursors. In addition, immunological responses may also have a direct impact on gametocytogenesis [8].

Thus, those with one negative allele (FY*B/FY*B^ES^ and FY*A/FY*B^ES^) might increase the ability of the host to develop immune response, and thereby limiting subsequent infections. This is potentially evidenced by the previous study demonstrated that the antibody responses against the blood stage antigens blocked the transmission of *P. vivax* to mosquitoes [38]. It could be due to the issue of liability of gametocytes, which can be affected by the host immune response against malaria parasites as explained by Vallejo et al. who detailed that high levels of cytokines IL-10, IFN-γ and TNF were correlated with low parasite infectivity of mosquitoes [7]. These plausible explanations lead to speculate that differences in mosquito infection depending on Duffy antigen polymorphism could be related with immune responses, however this study did not determine this.

Despite having greater rates of qualitative mosquito infection, individuals with homozygous Duffy positive blood group (TCT/TCT) exhibited a lower oocyst density per dissected mosquitoes which needs future investigation. This contradicting observation might be due to the difference in gametocyte maturation states and sex ratio [7] which were not examined in the present study. Moreover, the higher mean of oocyst load observed in membrane feeding assays performed using blood from FY*B/FY*BES genotyped individuals, here may be due in particular to the fact that the FY*B/FY*BES had higher parasite levels, as the current study and previous evidence has shown [39].

## Limitations of this study

This study failed to consider the effect of parasite, mosquito and human factors other than Duffy antigen polymorphism which could affect susceptibility of *Anopheles* mosquitoes. In addition, due to the small number of cases, it cannot affirm that the presence of the mutation in the FY gene can confer a degree of protection against the infection of mosquitoes by *P. vivax*.

## Conclusion

The infectiousness of *P. vivax*-infected patients was not significantly influenced by the Duffy blood group genotype of the patients. It is crucial to conduct additional research using a large sample size and a longitudinal study to evaluate the variations in mosquito infection and oocyst density according to Duffy antigen polymorphism.

## Data Availability

All relevant data are within the manuscript. The data that support the findings of this study are available from corresponding author on reasonable request.
